# Identification of CSRP1 as novel biomarker for hormone-sensitive prostate cancer by the combination of clinical and functional research

**DOI:** 10.1186/s12935-025-03708-y

**Published:** 2025-02-24

**Authors:** Chenxi Pan, Wei Wang, Yi He, Bo Yang

**Affiliations:** 1 State Key Laboratory of Fine Chemicals, Frontiers Science Center for Smart Materials Oriented Chemical Engineering, School of Bioengineering, Dalian University of Technology, No.2 Linggong Road, Dalian, 116023 PR China; 2 Department of Urology, The Second Hospital of Dalian Medical University, No.467 Zhongshan Road, Dalian, 116023 PR China

**Keywords:** Hormone-sensitive prostate cancer, Cysteine- and glycine-rich protein 1, WGCNA, Proteomics, Nomogram

## Abstract

Prostate cancer (PCa) ranks as the second most common malignancy and the fifth leading cause of cancer-related deaths among men. A critical challenge lies in accurately identifying those patients at high risk for transitioning rapidly from hormone-sensitive PCa (HSPC) to lethal castration-resistant PCa (CRPC). In our study, we employed a multiomics approach involving bioinformatics analysis on datasets GSE2443 and GSE35988, along with proteomics studies, to discover that cysteine- and glycine-rich protein 1 (CSRP1) expression significantly impacts the progression of HSPC. This hypothesis was substantiated through experiments using PC3 and LNCaP prostate cancer cells, where we conducted scratch assays and apoptosis assays, all of which confirmed CSRP1’s role in suppressing tumor growth. Furthermore, we elucidated the inhibitory effect of CSRP1 on tumors by performing xenograft experiments on castrated mice models. To solidify these findings in a clinical context, we constructed a nomogram model integrating CSRP1’s immunohistochemistry data and clinical parameters from an actual patient cohort with HSPC. This model revealed that low CSRP1 expression indeed promotes the advancement towards CRPC. In conclusion, the level of CSRP1 expression can serve as a valuable biomarker for clinicians to predict disease progression in their patients. It has the potential to guide personalized clinical management and decision-making strategies, thereby contributing to more effective and targeted treatment approaches for HSPC patients.

**Supplementary Information:**

The online version contains supplementary material available at 10.1186/s12935-025-03708-y.

## Introduction

Prostate cancer (PCa) stands as the second most frequent malignancy affecting men worldwide and is the fifth leading cause of cancer-related deaths [1]. The advent of prostate-specific antigen (PSA) screening, coupled with an aging population, has led to a rise in PCa incidence and mortality rates in China since 2012 [2]. Notably, locally advanced PCa and metastatic PCa cases carry significantly higher 10- and 15-year mortality risks compared to other PCa categories. Initial treatment regimens for patients with locally advanced or metastatic PCa often include Androgen Deprivation Therapy (ADT) combined with androgen receptor blockade. This combination therapy frequently proves efficacious initially [3]; however, a major challenge lies in the almost inevitable progression of hormone-sensitive prostate cancers (HSPC) to castration-resistant prostate cancers (CRPC). Within five years, virtually all HSPCs develop resistance to ADT, resulting in a grim prognosis where only a minority approximately 5 to 10% of patients survive for ten years following the initiation of ADT [4].

Given the heterogeneity of PCa, its clinical presentation ranges from indolent to aggressive subtypes, making it a disease with a highly diverse spectrum. The transition period from HSPC to CRPC is notably variable across patients, yet this aspect has not been extensively researched. A series of pivotal phase-III randomized controlled trials such as CHAARTED and LATITUDE have shown that identifying whether a patient with HSPC is likely to develop CRPC in either a short or long time frame can significantly influence treatment strategies [5, 6]. These studies suggest that early detection and timely intervention can be helpful when it comes to managing these patients. Thus, there is an urgent need for accurate predictive methods to identify which patients with HSPC re at risk of progressing to CRPC within a shorter duration. This would enable clinicians to devise more tailored follow-up plans and optimize therapeutic regimens, ultimately improving patient outcomes and survival rates.

Cysteine- and glycine-rich proteins (CSRPs) are integral members of the LIM domain protein superfamily, which is highly conserved across both vertebrate and invertebrate species. The LIM domain, a hallmark of these proteins, is known to participate in an array of cellular processes, from gene regulation to cytoskeletal organization [7]. Within vertebrates, there are three recognized CSRP family members: CSRP1, CSRP2, and CSRP3/MLP, as reported in studies [8, 9]. In recent years, research on CSRP1’s role in cancer has gained momentum, with its strongest associations being found in urogenital tumors. Studies have shown that CSRP1 is closely related to adrenocortical carcinoma [10], the progression from prostate hyperplasia to prostate cancer [11, 12], bladder cancer [13], and kidney renal papillary cell carcinoma [14]. However, the contemporary clinical challenge lies not only in understanding tumor onset but more critically in accurately identifying disease status during treatment. Among these challenges, the transition of prostate cancer from HSPC to CRPC stages remains one of the most difficult to predict and manage. While research on CSRP1’s involvement in various cancers is advancing, its specific role in HSPC has yet to be fully explored and constitutes a significant gap in current knowledge, necessitating further investigation.

In the present study, we initiated by uncovering a significant correlation between CSRP1 expression and the progression of HSPC to CRPC through an analysis of our proteomic data and publicly available databases. Subsequently, we employed both in vitro cell line models and in vivo experimental systems to elucidate the functional role of CSRP1 in this process. Our research efforts culminated in the development of a predictive model that utilizes immunohistochemical staining results for CSRP1 from our clinical patient cohort, enabling us to identify patients with HSPC who are more likely to progress to CRPC. This model represents a crucial step forward in enhancing our understanding of disease progression and could potentially guide personalized therapeutic strategies for patients with prostate cancer.

## Materials and methods

### Patients and tissues

In this retrospective research, we enrolled a total of 178 patients diagnosed with HSPC and possessing complete follow-up data, from January 2016 to July 2023 at the Second Hospital of Dalian Medical University. The study protocol was meticulously reviewed and received ethical approval by the Ethics Committee of the Second Hospital of Dalian Medical University, ensuring compliance with all relevant regulations and guidelines. Furthermore, our study has been registered in the Chinese Clinical Trial Register under registration number ChiCTR2100054836 to maintain transparency and adherence to international standards for clinical research. Prior to their inclusion in the study, each patient provided written informed consent, thereby confirming their voluntary participation and understanding of the study’s purpose and procedures.

All patients with prostate adenocarcinoma newly confirmed by pathology, were diagnosed with locally advanced or metastatic disease, and received ADT (LHRH agonists) combined with anti-androgen as initial and only therapy before CRPC progression. Variables of patients included age at PCa diagnosis (years), PSA at PCa diagnosis (ng/ml), clinical TNM staging information, biopsy Gleason sum, progression-free survival (PFS, months), which is identified from the date of therapy to the date of CRPC advancement. All pathological diagnoses were confirmed by 2 pathologists independently under the World Health Organization (WHO). Patients who advanced to CPRC fulfilled the following requirements: serum testosterone level under 50 ng/dl (1.7 nmol/L) plus one of the following types of progression: (1) PSA progression: PSA level above 2.0 ng/mL, interval 1 week, three times higher than the baseline level > 50%; (2) Radiological progression: the appearance of new lesions—either two or more new bone lesions on bone scan or a soft tissue lesion using the Response Evaluation Criteria in Solid Tumours.

### Public data sources

In this study, we integrated data from multiple sources to identify genes potentially associated with the progression of HSPC to CRPC. To accomplish this, bulk RNA sequencing data for HSPC and CRPC samples were extracted from two public datasets: GSE2443, which comprised 20 samples, and GSE35988, containing 76 samples.

To investigate the expression pattern of CSRP1 and its potential link to disease-free survival, we obtained gene expression and survival data from TCGA-PRAD dataset, consisting of a total of 492 cases. This information was then visualized and analyzed using the GEPIA2 web-based tool (accessible at http://gepia2.cancer-pku.cn/#index), enabling us to explore the relationship between CSRP1 expression levels and patient outcomes in a comprehensive manner.

### Bioinformatics analysis

The limma(linear models for microarray data, 10.1093/nar/gkv007) is a widely used statistical approach based on generalized linear models for identifying differentially expressed genes in high-throughput transcriptomic studies. In this study, we employed the R package limma (version 3.40.6) to perform differential expression analysis. Specifically, we applied the lmFit function to conduct multiple linear regression on our obtained gene expression profile dataset, comparing different experimental groups against control groups. Subsequently, we utilized the eBays function to calculate moderated t-statistics and F-statistics. This involves empirical Bayes moderation, which shrinks the standard errors towards a common value, thereby improving the accuracy and stability of the significance tests. This process also generates log2 fold change values (log2FC) for each gene, representing the relative difference in expression between conditions. Finally, we determined significant differences by setting a threshold where genes were considered differentially expressed if they met both criteria: *P* < 0.05 and|log2FC| > 1.5. These stringent cutoffs helped us to identify genes that exhibit robust and biologically meaningful changes in expression during the progression from HSPC to CRPC.

Initially, we employed the differentially expressed genes (DEGs) obtained from GSE35988 dataset (filtered with FDR < 0.05 and|log2FC| ≥ 1.5) to construct a scale-free co-expression network using WGCNA. The process began by calculating the Pearson’s correlation coefficients for all pairs of genes followed by applying an average linkage method. Subsequently, a weighted adjacency matrix was formulated based on these correlations through a power function: A_mn =|C_mn|^β, where C_mn is the Pearson’s correlation between Gene_m and Gene_n, and A_mn represents their adjacency in the network. The soft-thresholding parameter β was chosen to accentuate strong correlations while downplaying weak ones. In this case, a power of 16 was selected to optimize the scale-free topology criterion. The adjacency matrix was then converted into a Topological Overlap Matrix (TOM), which serves as a measure of the interconnectedness of each gene within the network. The corresponding dissimilarity (1-TOM) was calculated to facilitate hierarchical clustering. Average linkage hierarchical clustering was conducted based on the TOM-based dissimilarity metric, grouping genes with similar expression patterns into distinct modules. We set the minimum module size at 50 genes, and the sensitivity threshold to 3. Further refinement involved calculating the dissimilarities among module eigengenes and cutting the module dendrogram accordingly, merging some modules that were closely related. Additionally, any modules with a distance less than 0.25 were combined, resulting in a total of 7 co-expression modules. It should be noted that genes not assigned to any module were grouped into a “grey” module.

To gain functional insights into these modules, KEGG pathway and GO enrichment analyses were carried out using Cytoscape software (version 3.9.1) equipped with the ClueGO plugin (version 2.5.9). This allowed us to identify enriched biological pathways and molecular functions associated with the identified gene modules.

### Proteomics sample preparation and data processing

At the times of CRPC diagnosis, three patients received radical prostatectomy, of whom the therapy-naïve biopsies and CRPC specimens were collected for proteomic analysis using the PCT-Pulse DIA technology, with one patient’s undergoing technical duplication. Briefly, formalin-fixed paraffin-embedded (FFPE) tissue samples were punched (diameter 1 mm) from FFPE blocks at the histopathological areas with the primary Gleason pattern marked by the same pathologist. Approximately 0.2 mg FFPE punches were dewaxed with heptane, hydrated with ethanol, and then underwent acidic hydrolysis by 0.1% formic acid (FA, Thermo Fisher Scientific, USA) and basic hydrolysis by 0.1 M Tris-HCl (pH = 10.0). Samples were next lysed using a 6 M urea/2 M thiourea buffer (Sigma, USA), reduced by tris (2 carboxyethyl) phosphine (Sigma, USA), and alkylated by iodoacetamide (Sigma, USA). The lysates were then digested using PCT by a mix of Ly s-C and trypsin (Hualishi Tech. Ltd., China). Finally, the PCT-assisted digestion reaction was stopped by trifluoroacetic acid and cleaned by C18.

A total of 400 ng peptides were injected and separated along a 45 min liquid chromatography gradient (from 3 to 28% buffer B-see below for its composition) at a flow rate of 300 nL/min (precolumn: 3 μm, 100 Å, 20 mm × 75 μm i.d.; analytical column: 1.9 μm, 120 Å, 150 mm × 75 μm i.d.). Buffer A was mass spectrometry-grade water containing 2% acetonitrile and 0.1% FA; buffer B was acetonitrile containing 2% H2O and 0.1% FA. The peptides were then analyzed by a Q Exactive HF hybrid Quadrupole-Orbitrap (Thermo Fisher Scientific, USA) using the PulseDIA mode with four pulses. Then, PulseDIA files were analyzed using Spectronaut with default settings, based on our established library comprised 143,347 peptide precursors, 115,257 modified peptides, and 9,644 proteins [15].

### Immunohistochemistry (IHC) analysis

Following proteomic and bioinformatic analyses, we sought to validate the correlation between CSRP1 expression and patient prognosis using IHC on pre-ADT biopsy specimens from an additional 175 cases. The FFPE tissue samples were sectioned at a thickness of 4 micrometers and affixed onto poly-L-lysine-coated slides. The slides underwent dewaxing in xylene and rehydration through graded alcohol series. To retrieve antigenicity, sections were subjected to heat-induced epitope retrieval in a citrate buffer solution (0.01 mol/l citric acid, pH 6.0) for 20 min at 95 °C. Subsequently, endogenous peroxidase activity was blocked by immersing the sections in PBS containing 3% H2O2 for 10 min at room temperature. To reduce nonspecific binding, the sections were incubated with 10% normal goat serum in PBS for 30 min at room temperature. They were then treated overnight at 4 °C with a rabbit polyclonal anti-CSRP1 antibody diluted at 1:1000 (obtained from Abcam). After thorough washing with PBS, the sections were incubated with biotinylated Goat anti-rabbit IgG secondary antibody for 20 min at room temperature, followed by treatment with a 3,30-diaminobenzidine (DAB) chromogen solution for five minutes to visualize the bound primary antibody. Finally, the sections were counterstained with hematoxylin for six minutes to stain the nuclei. The assessment of CSRP1 immunostaining was carried out as follows:

1. For positive cell percentage scoring: scores were assigned as 0 (≤ 5%), 1 (6–25%), 2 (26–50%), 3 (51–75%), or 4 (> 75%).
2. Based on staining intensity: scores ranged from 0 (no coloration), 1 (light yellow), 2 (brown), to 3 (yellow-brown).

The final IHC score for each case was calculated as the product of the two individual scores, giving a range from 0 to 12. All evaluations of the immunostained sections were performed blindly without knowledge of the patients’ clinicopathological data to ensure objectivity and accuracy of the results.

### Establishment and validation of predicting nomogram for the HSPC progression

The R package “survival” package was utilized to integrate and analyze the patient data, which included survival time, survival status, and various clinicopathological characteristics. To assess the independent prognostic significance of these factors in the training dataset, a multivariate Cox regression analysis was conducted. Building upon the results from both univariate and multivariate Cox proportional hazards analyses, we constructed a nomogram using the “RMS” package that predicts the probability of disease progression at 6, 12, 18, and 24 months. This nomogram visually represents the contribution of each risk factor with points assigned to each factor; by summing up these points, an individual patient’s prognosis risk can be estimated. During validation of this nomogram, the total points for every patient within the validation cohort were calculated according to the established nomogram model. The predictive accuracy of the nomogram was evaluated using two metrics: the C-index, which measures the ability of the model to correctly rank patients based on their actual outcomes, and the ROC curve, which reflects the diagnostic power across different threshold probabilities. Moreover, calibration plots were generated to evaluate the agreement between the predicted and observed risks of disease progression, ensuring the nomogram’s predictive precision. Additionally, decision curve analysis (DCA) was employed to quantify the net benefit of our nomogram in clinical practice, thereby assessing its overall utility and practical application value.

### Cell culture and transfections

PC3 and LNCaP prostate cancer cells were sourced from the American Type Culture Collection (ATCC) and maintained in RPMI-1640 medium (Invitrogen, Carlsbad, CA) enriched with 10% fetal bovine serum (FBS; Invitrogen), 100 U/mL penicillin, and 100 mg/mL streptomycin at a standard culture condition of 37 °C under a humidified atmosphere containing 5% CO2. To simulate the ADT typically administered to PCa patients, cells were initially cultured in complete medium for 24 h. Subsequently, PC3 and LNCaP were exposed to androgen-deprivation media (ADM) consisting of charcoal-stripped serum (C-S serum, Gibco) devoid of androgens and supplemented with 10 µM and 6 µM Bicalutamide (Beyotime Biotechnology), respectively. For the overexpression of CSRP1, 3 × 10^6^ PC3 or LNCaP cells were transfected using 2 µg of pcDNA3.1-CSRP1 plasmid or an empty vector as a control, utilizing 6 µL Lipofectamine 2000 (Invitrogen, Carlsbad, CA, USA) according to manufacturer’s guidelines. After two days of post-transfection incubation, cells were reseeded into 10 cm dishes and allowed to grow for another two days. Thereafter, the selection process was initiated by adding complete culture medium containing 1000 µg/ml G418 (Sigma, St. Louis, MO) to the culture. After a 20 day period of selective pressure with G418, stable cell lines were established and designated as PC3/CSRP1 or LNCaP/CSRP1. The successful overexpression of CSRP1 in these cells was confirmed through quantitative Real-Time PCR analysis. A lentiviral shRNA vector targeting CSRP1 was generated by inserting stranded oligonucleotides (sh-CSRP1, forward sequence 5′- CCG GGC TTC CAT AAA TCC TGC TTC CCT CGA GGG AAG CAG GAT TTA TGG AAG CTT TTT G-3′) into TRC2-pLKO-puro Vector (Sigma-Aldrich). LNCaP cells were infected with the CSRP1 shRNA vectors according to the manufacturer’s protocol. After 5 days of selection in 1640 medium containing 4 µg/ml puromycin, stable CSRP1 down-regulated colonies were isolated and designated as LNCaP-CSRP1. The transfection efficiency was confirmed by quantitative real-time PCR analysis.

### Quantitative Real-Time PCR

The RNA isoPlus^®^ Reagent Kit (Takara Biotechnology) was used to extract RNA from PCa cells according to the manufacturer’s instructions. The PrimeScript^®^ RT Reagent Pack (Takara, Shiga, Japan) was used to convert RNA into cDNA. The cDNA was amplified using the SYBR^®^ Premix Ex Taq™ Unit under the 7500 Continuous PCR Framework (Applied Biosystems, Thermo, US). The cycling conditions were as follows: The data was analyzed using the comparative Ct method, with GAPDH serving as the loading control for the target genes throughout forty cycles of 95°C for 30 s and 60°C for 34 s. The preliminary sequences were as follows: CSRP1 (forward: 5’- TGCCGAAGAGGTTCAGTGC-3’, reverse: 5’-AGCAGGACTTGCAGTAAATCTC-3’); GAPDH (forward: 5’-GCACCGTCAAGGCTGAGAAC-3’, reverse: 5’-TGGTGAAGACGCCAGTGGA-3’).

### Flow cytometry analysis of cell apoptosis

Cells were seeded in six-well plates at a density of 1 × 10^5^ cells per well, with each well containing 2 milliliters of growth medium. After allowing the cells to adhere and grow for 24 h, they were subjected to ADM for another 24-hour period to simulate the conditions that prostate cancer cells encounter during hormonal therapy. Upon completion of the ADM treatment, the cells were carefully collected and rinsed thoroughly with PBS to remove any residual media or debris. The cells were then resuspended in 300 µl of binding buffer to prepare them for flow cytometry analysis. To stain the cells for apoptosis detection, 5 microliters of PI was added to each cell suspension, which was then incubated in the dark for 15 min to allow PI to bind DNA content, thereby enabling discrimination between viable, apoptotic, and necrotic cells based on their fluorescence intensity. Finally, the stained cells were analyzed using a BD FACSVerse flow cytometer to quantify and characterize the extent of apoptosis within the treated cell population. This instrument uses laser-based technology to measure the fluorescence emitted by PI-labeled cells, providing data on cellular DNA content and thus allowing for the determination of the proportion of cells undergoing apoptosis in response to the ADM treatment.

### Migration assay

Wound healing assay was used to detect the ability of migration of cell lines. The cells with concentration of 1 × 10^4^/ml were cultured in 6-well plate at 37 °C with 5% CO2 for 24 h and grown until 80% confluent. was added to culture for 24 h. A straight line scratch was made on the cells using a sterile 20 µL disposable serological pipette. The cells were washed with 1 ml median to remove debris and smooth the edge of the scratch and cultured in median without FBS for 12 h. Images of the cell proliferation were taken using a microscope.

### Animal study

The animal care and experimentation in this study strictly adhered to the ARRIVE (Animal Research: Reporting of In Vivo Experiments) guidelines, as well as the U.K. Animals (Scientific Procedures) Act 1986 and its associated guidelines, along with the EU Directive 2010/63/EU for animal experiments, ensuring the highest standards of humane treatment. All procedures involving animals were meticulously reviewed and approved by the ethics committee of Dalian Medical University, under Permit Number: AEE23112. For this research, male BALB/c nude mice aged 4–6 weeks old (with a body weight range of 20 ± 2 grams, total *n* = 10) were housed in isolated ventilated cages within a specific pathogen-free environment. The housing conditions were carefully controlled for temperature and humidity, and maintained a 12-hour dark/light cycle to mimic their natural circadian rhythm. These mice had unrestricted access to tap water and standard pellet food to ensure optimal health and wellbeing. Daily monitoring was conducted to assess their overall health status throughout the course of the experiment. LNCaP/pcDNA3.1 cells or LNCaP/CSRP1 cells (2 × 10^6^/0.1 mL) suspended in 50% matrigel in RPMI 1640 were injected s.c. into the right flank of the mice. After 5 weeks, all mice with well-established tumors (0.6–1.0 cm long and 0.6–1.0 cm wide) were anesthetized with intraperitoneal 75 mg/kg of ketamine and 1 mg/kg of medetomidine, and took orchidectomy. Analgesia was provided by subcutaneous administration of 0.05 mg/kg of buprenorphine before and after the operations. Animals were monitored daily for signs of infection, pain, and discomfort. Tumor sizes were monitored every day by calliper and tumor volume were calculated according to the formula: S^2^ × L/2 (S = shorter diameter, L = longer diameter of the tumor). In this study, when tumors in castrated mice started regrowth and reached 100% of their initial size, they were considered to be CRPC stage. Once in CRPC stage, tumors were observed by Super Nova^®^ PET/CT (SNPC-103, Pingsheng Medical Technology (Kunshan) Co., Ltd. China), then mice were euthanised with carbon dioxide.

### Statistical analysis

The optimal cutoff values for age and CSRP1 expression scores were calculated using the X-tile software version 3.6.1, a tool developed by Yale University. All statistical analyses in this study were executed using SPSS Statistical Package version 24.0 (SPSS Inc., USA). Categorical variables are presented as frequencies and proportions. The hazard ratios (HRs) along with their corresponding 95% CI were derived through multivariate Cox regression analysis, leveraging the “survival” package in R for survival data analysis. For decision curve analysis (DCA), we employed the “ggDCA” R package to assess the clinical utility of our model predictions. ROC analysis was conducted utilizing the R software package pROC (version 1.17.0.1) to calculate the AUC, which measures the discriminatory power of the risk score model. This was done specifically by analyzing the patients’ follow-up duration and risk score at different time points (6, 12, 18, and 24 months) and employing the ROC function from the “pROC” package. In all inferential procedures, statistical significance was set at a P-value threshold of less than 0.05.

## Results

### Integrated screening for CRPC-associated genes

**Fig. 1:**
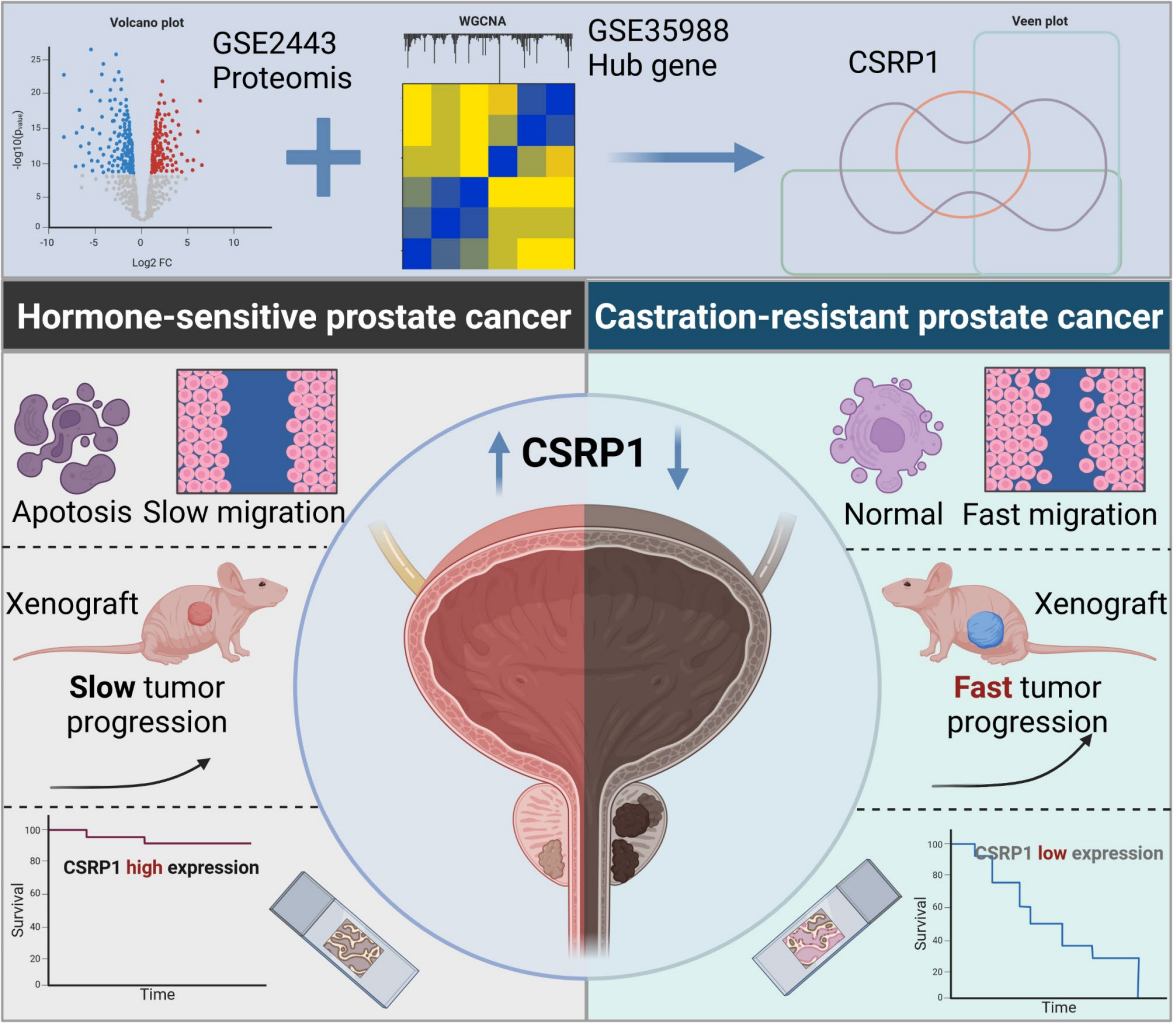
study design and workflow of the study. Created from Biorender.com

In order to delve into the molecular mechanisms underlying the progression of HSPC to CRPC, we performed proteomic analysis on three pairs of CRPC surgical specimens and their corresponding therapy-naïve HSPC biopsy samples, obtained from three distinct patients (labeled as CR1, CR2, and CR3). The clinicopathological details of these patients are summarized in Supplementary Table 1. Through our extensive proteomic examination, we identified a total of 7,393 protein groups and 7,298 proteotypic proteins across the six analyzed samples. These comprehensive datasets provide valuable insights into the proteome changes that occur during the transition from HSPC to CRPC. The mass spectrometry-based proteomics data generated in this study have been deposited in the public domain at the ProteomeXchange Consortium (accessible through the URL: http://proteomecentral.proteomexchange.org) via the iProX partner repository (PMID: 30252093). The dataset can be accessed using the unique identifier IPX0007011000, enabling researchers worldwide to review, validate, and build upon our findings (Fig. 1).

**Fig. 2:**
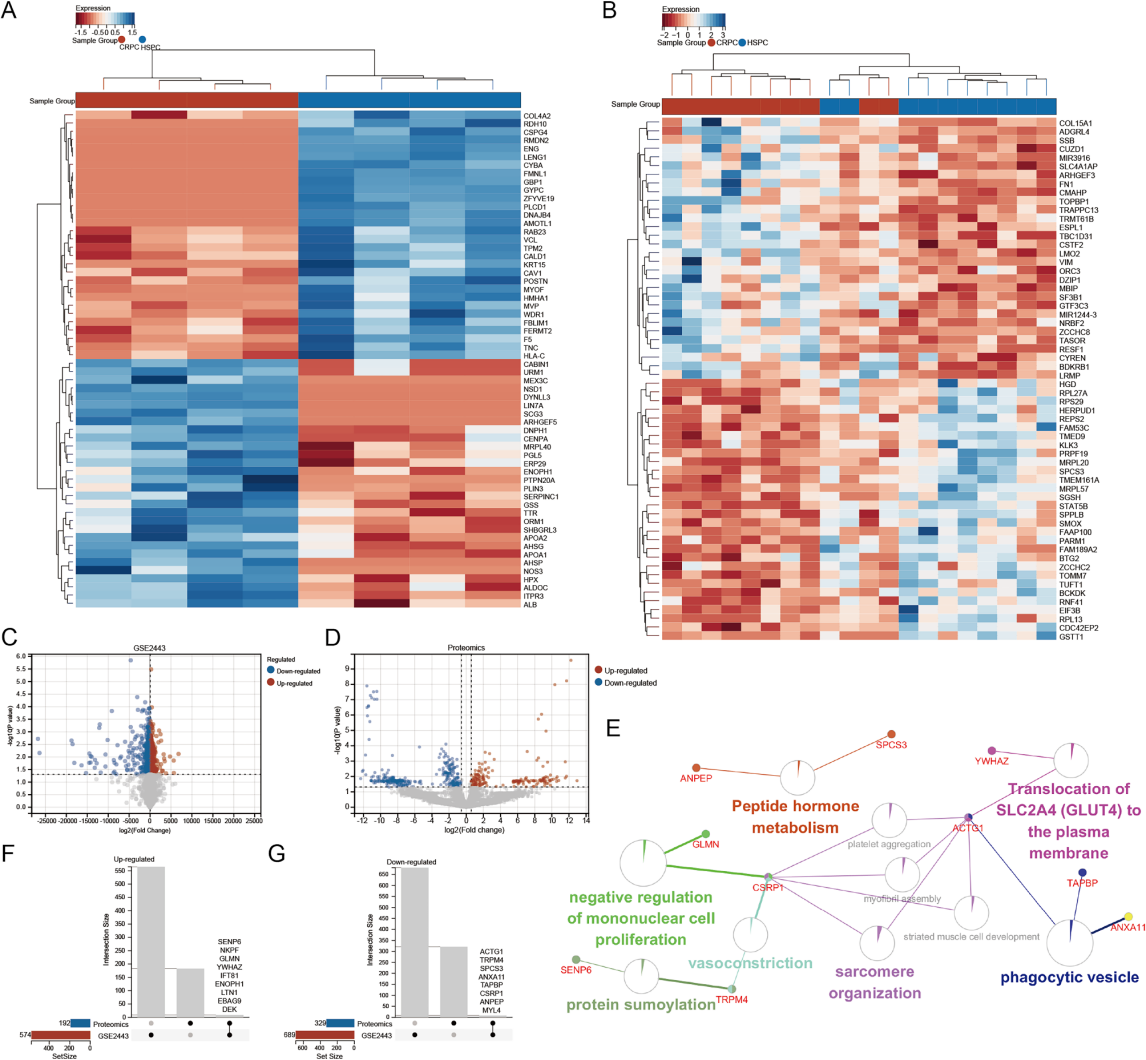
Multiomics screening for differentially expressed molecules and pathways. (**A**) Heat map showing proteomics differential protein expression. (**B**) Heat map showing differential gene expression of GSE2443. (**C**) The volcano map shows the expression of differential genes in GSE2443. (**D**) The volcano map shows the expression of differential proteins in proteomiccs. Blue dots indicate down-regulated and red dots indicate up-regulated. (**E**) Pathway analysis of a set of differential expressions of GSE2443 and proteomics. (**F**) Up-regulated molecule of GSE2443 and proteomics. (**G**) Down-regulated molecule of GSE2443 and proteomics

Using the “limma” R package, we normalized expression data from datasets GSE2443 and proteomics, and identified 1263 and 521 differential expression molecules between localized CRPC and HSPC tumors respectively, with a cut-off of *p* < 0.05 (Fig. 2A-D). Among the differential genes of GSE2443 and proteomics, there were 9 up-regulated genes and 8 down-regulated genes. the overlapping differential gene pathways are enriched in translocation of solute carrier family 2 (SLC2A4) to the plasma membrane, peptide hormone metabolism, negative regulation of mononuclear cell proliferation, vasoconstriction, sarcomere organization, and phagoacytic vesicle. Core genes include CSRP1 and actin gamma 1 (ACTG1) (Fig. 2E).

### Weighted Gene Co-expression Network Analysis identification of key modules

**Fig. 3:**
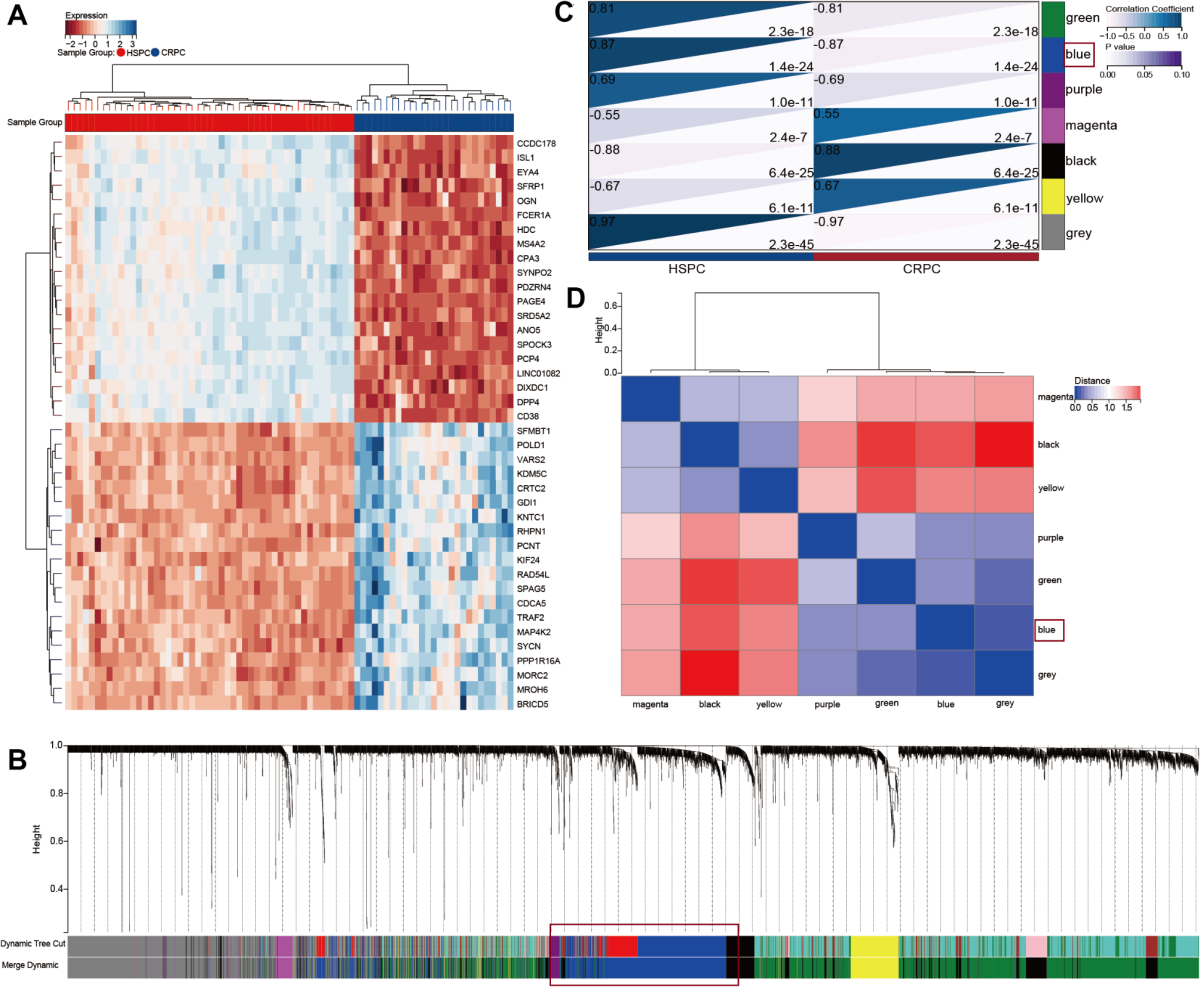
The WGCNA algorithm identify the hub gene associated with HSPC progression. (**A**) The heat map shows the top 40 differentially expressed genes. (**B**) Dendrogram of all DEGs clustered based on 1-TOM. (**C**) Correlation heatmap between module eigengenes and CRPC. (**D**) Vector clustering of all feature modules

Weighted Gene Co-expression Network Analysis (WGCNA) was performed using the expression profiles of DEGs in the GSE35988 cohort (Supplementary Fig. 1A). And, we used heat maps to visualize the first 30 differentially expressed genes (Fig. 3A). The higher the singed R2, the closer the network is to the distribution of a scale free network. When signed R2 is greater than 0.85, the network already conforms to the distribution of the network. Therefore, the RsquaredCut parameter in the soft threshold is computed in the WGCNA package, with a default value of 0.85. We then repeatedly confirmed and choose 16 as the soft threshold power in the GSE35988-DEGs cohort (Supplementary Fig. 1B-C). Subsequently, dynamic module identification was performed in the different cohorts, with the number of genes per module not less than 50 (Fig. 3B-C). Cluster plot analysis of the relationship between different modules (Fig. 3D).

### Low expression of CSRP1 is the factor that leads to the progression of HSPC to CRPC

**Fig. 4:**
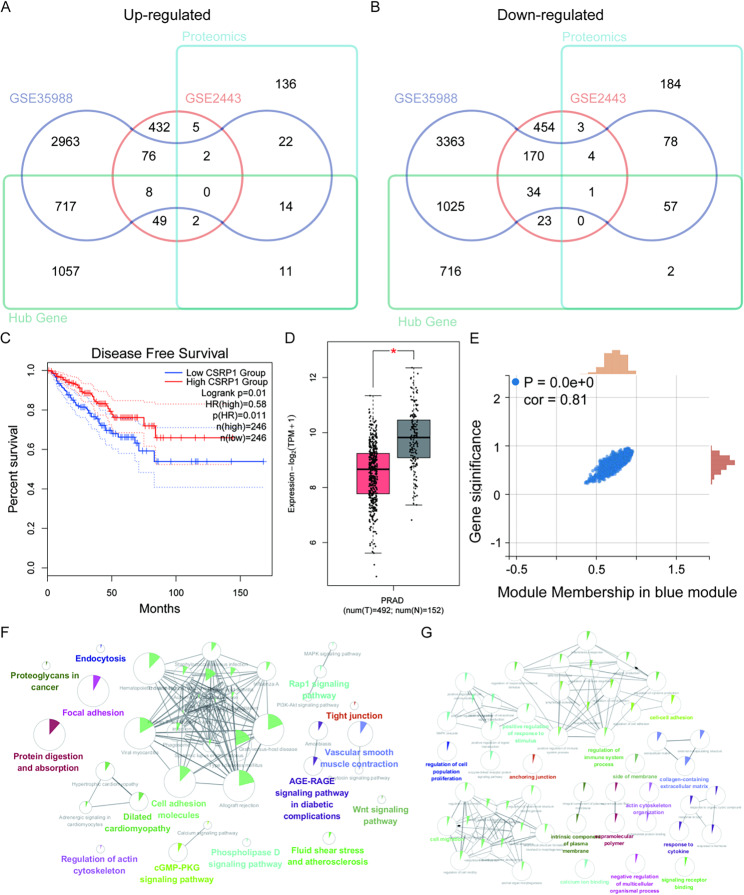
Determination of the characteristic molecule CSRP1. (**A**) The up-regulated molecules of GSE35988, GSE2443 and proteomics were shown in Veen diagram. (**B**) The down-regulated molecules of GSE35988, GSE2443, and proteomics were shown in Veen diagram. (**C**) Relationship between disease free survival and CSRP1 expression in patients with TCGA-PRAD. (**D**) Expression of CSRP1 in TCGA-PRAD tissue and normal tissue. (**E**) Correlation between CSRP1 and blue module. (**F**) KEGG analysis of blue module. (**G**) GO analysis of blue module

The intersection of upregulated differential genes of GSE2443, GSE35988 and proteomics was 0 (Fig. 4A). The intersection of down-regulated differential genes of GSE2443, GSE35988 and proteomics was CSRP1(Fig. 4B). At the same time, we found through The Cancer Genome Atlas Prostate Adenocarcinoma (TCGA-PRAD) database demonstrated that the disease free survival of the Low CSRP1 group was worse than that of the High CSRP1 group (Fig. 4C). In TCGA, the expression of CSRP1 in prostate tumor tissues was significantly lower than that in normal tissues (Fig. 4D). We can also see that CSRP1 gene is very related to HSPC and CRPC (*p* = 0.0E + 0; *r* = 0.81) (Fig. 4E). Subsequently, Kyoto Encyclopedia of Genes and Genomes (KEGG) pathway analysis and Gene ontology (GO) analysis were performed on the genes of the blue module. In the KEGG analysis, we can see that the main genes are associated with cell adhesion molecules (Fig. 4F). In the GO analysis, we can see that the main genes are related to regulation of cell motility (Fig. 4G).

### CSRP1 suppressed the LNCaP cells growth and sensitizes cells to androgen-deprivation therapy in vitro and in vivo

**Fig. 5:**
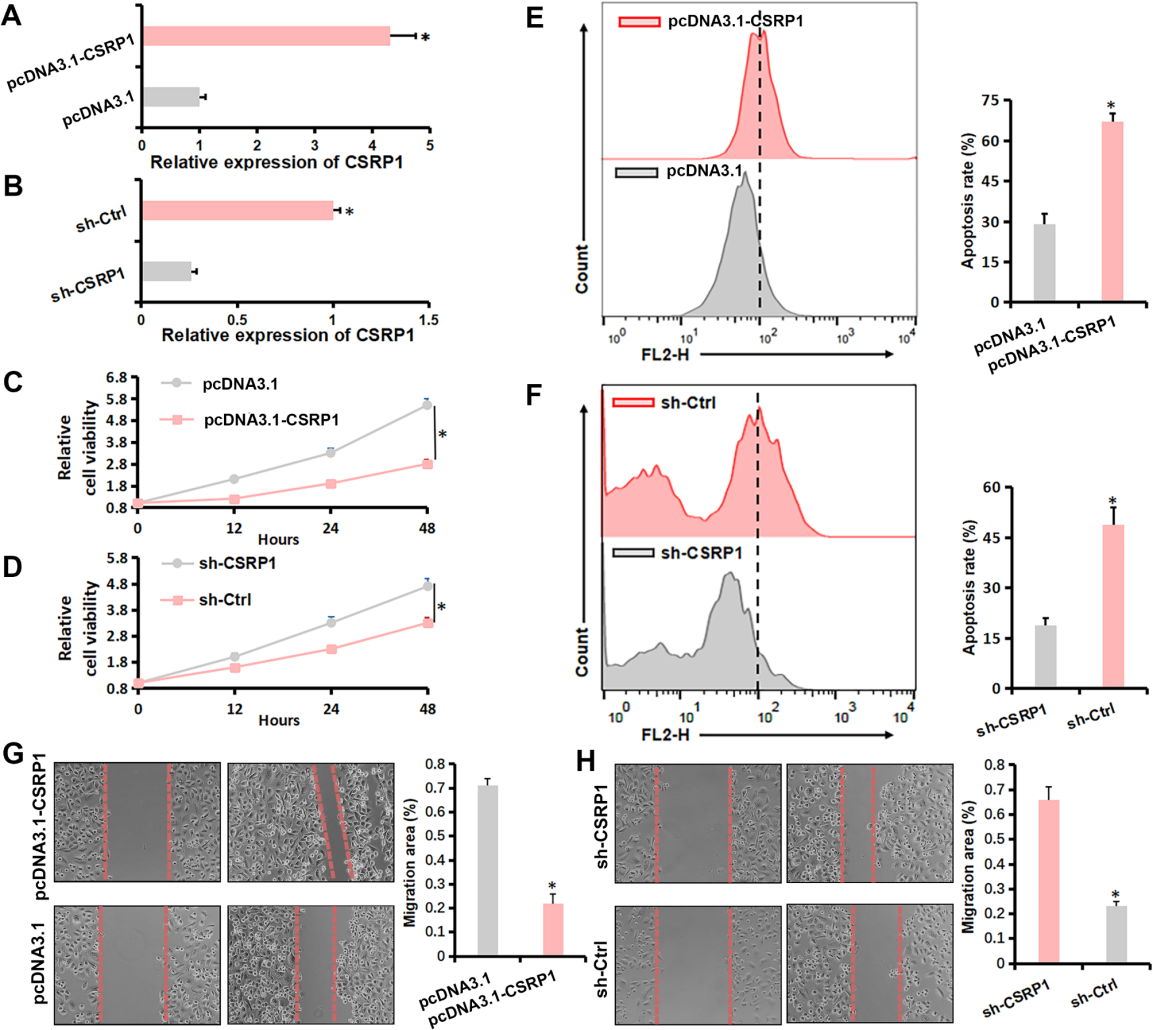
Effect of CSRP1 expression level on the malignancy of PCa cells. (**A**) Stable CSRP1 overexpression using pcDNA3.1-CSRP1 was confirmed by qRT-PCR in PC3 cells; (**B**) Stable CSRP1 knockdown was confirmed by qRT-PCR in LNCaP cells; (**C**) Effect of CSRP1 overexpression on the viability of PC3 cells was detected by CCK-8 assays; (**D**) Effect of CSRP1 knockdown on the viability of LNCaP cells was detected by CCK-8 assays; (**E**) Cell apoptosis of PC3 cells cultured in the ADM containing charcoal-stripped serum and Bicalutamide (10 µM) were analyzed via flow cytometry assay. (**F**) Cell apoptosis of LNCaP cells cultured in the ADM containing charcoal-stripped serum and Bicalutamide (6 µM) were analyzed via flow cytometry assay. (**G**) Effect of CSRP1 overexpression on the migration ability of PC3 cells detected via wound healing assay, magnification: 100×; (**H**) Effect of CSRP1 knockdown on the migration ability of LNCaP cells detected via wound healing assay, magnification: 100×. All data are presented as the mean ± SD for three independent experiments (* *P* < 0.05)

**Fig. 6:**
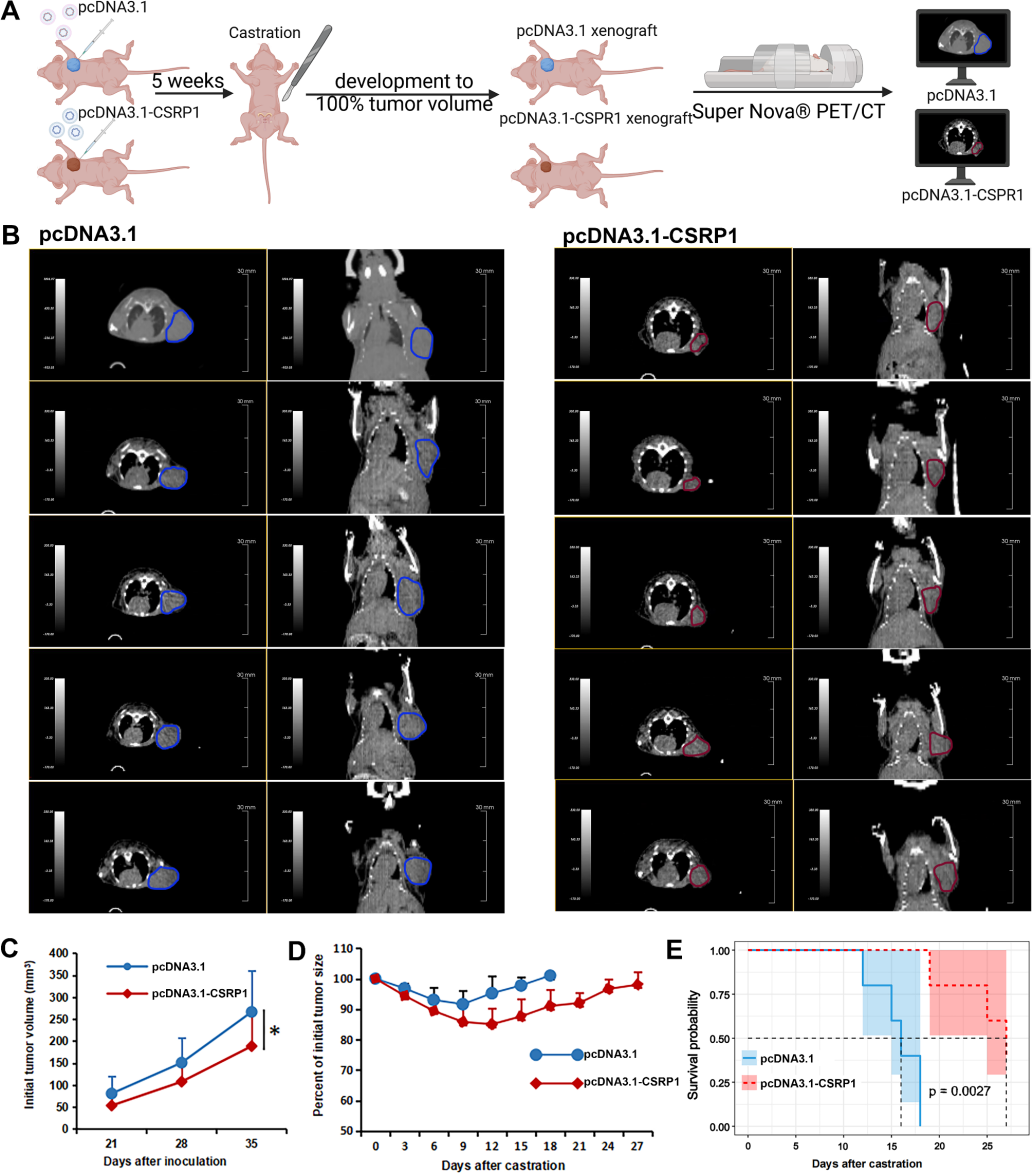
CSRP1 overexpression suppressed tumor growth and sensitized LNCaP tumor to androgen-deprivation therapy. (**A**) Schematic illustrating the LNCaP mouse xenograft experimental design. (**B**) CT imaging for CRPC tumors in two groups (*n* = 5). (**C**) Tumor growth curve before mice being castrated. (**D**) The percent change in volume for tumors in castrated mice bearing LNCaP xenograft. (**E**) Kaplan-Meier analysis of CRPC-free survival in mice according to CSRP1 expression (log-rank test: *p* = 0.0027). All data are presented as the mean ± SD for three independent experiments (* *P* < 0.05)

To validate the anti-oncogenic properties of CSRP1 in PCa, we generated PC3/CSRP1 (Fig. 5A), LNCaP-CSRP1 (Fig. 5B) and LNCaP/CSRP1 (Supplementary Fig. 1D) cells. Increased CSRP1 expression significantly inhibited the cell viability of PC3 cells (Fig. 5C), while CSRP1 knockdown significantly promoted the cell viability (Fig. 5D) of LNCaP cells. Cell apoptosis analysis showed that CSRP1 overexpression could significantly enhance the sensitivity to ADM condition in PC3 cells (Fig. 5E), and CSRP1 knockdown could significantly enhance the resistance to ADM condition in LNCaP cells (Fig. 5F). Furthermore, increased CSRP1 expression considerably reduced the migratory ability of PC3 cells (Fig. 5G), whereas CSRP1 knockdown greatly increased the migration ability of LNCaP cells (Fig. 5H). Studies in vivo demonstrated that CSRP1 overexpression considerably reduces the LNCaP tumor growth compared to control group (Fig. 6A, B). After 5 weeks, all the tumors reached a moderate size (0.6–1.0 cm wide and 0.6–1.0 cm long), the mice were surgically castrated. In all groups, the LNCaP tumors regressed initially in response to castration, but the tumors then progressed to androgen independence (Fig. 6C). Regrowth of the tumors started at 7, 7, 8, 10 and 10 days in the scramble group, and 10, 13, 13, 14 and 14 days after castration in CSRP1 group, respectively. In the current study, CRPC stage was defined as tumors in castrated mice started regrowth and reached 100% of their initial size. The time that it took for the tumors advanced to CRPC was 12, 15, 16, 18 and 18 days in the scramble group, and 19, 25, 27, > 27 and > 27 in the CSRP1 group, respectively (Fig. 6D). The results indicate that CSRP1 played anti-oncogene roles in PCa, suppressed the formation and growth of prostate tumors, and promoted their androgen dependence.

### CSRP1 could be a novel HSPC prognostic factor associated with castration-resistant conversion

**Fig. 7:**
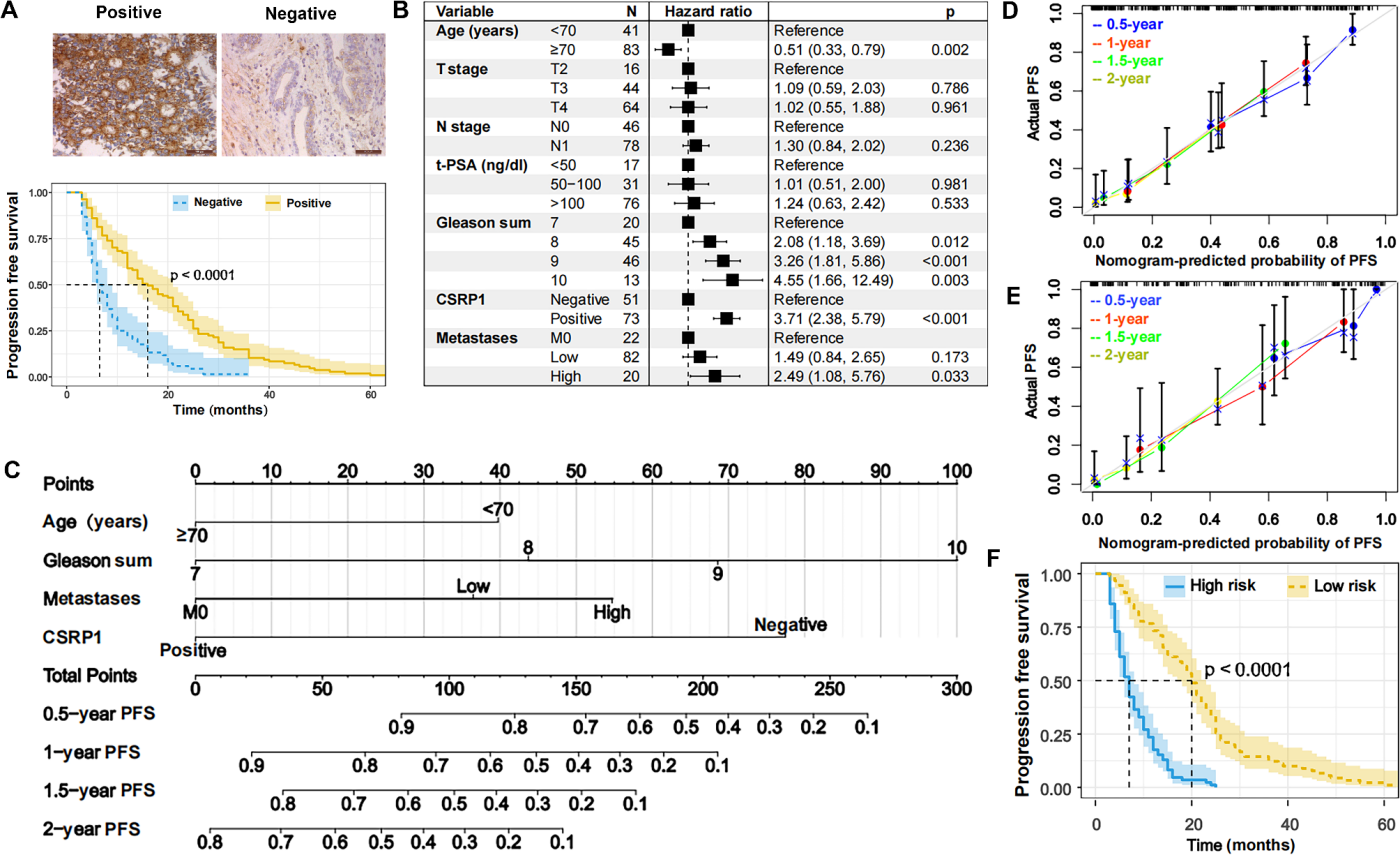
CSRP1 could be a novel HSPC prognostic factor. (**A**) Kaplan-Meier analysis of progression free survival (PFS) in HSPC patients according to CSRP1 expression in biopsy tumor (log-rank test: *p* = 0.0027). (**B**) Multivariable Cox regression analysis for independent factors of HSPC progression. (**C**) Nomogram based on age, Gleason sum, metastases and CSRP1 expression for predicting the 6-month, 12-month, 18-month and 24-month PFS. (**D**) Calibration plots for assessing the predictive accuracy of the nomogram in training and validation set, respectively. (**E**) Kaplan-Meier analysis of PFS based on nomogram correlated risk score in the whole patients set

In order to develop a model for predicting the HSPC progression, assisting clinicians in recommending personalized therapeutic guidance, we performed the immunohistochemistry (IHC) analysis of therapy-naïve biopsy specimens of 175 HSPC patients, using an immunoscore (range, 0 to 12) to quantify the CSRP1 protein expression. Using X-tile software v3.6.1 (Yale University), patients were split into positive (score > 4) and negative groups (score ≤ 4), with 61.14% (*n* = 107) in the positive group and 38.86% (*n* = 68) in the negative group (Table 1). Between the two groups, there was a statistically significant difference in prognosis (Fig. 7A).

**Table 1:** Representativeness of study participants

Characteristics	Categories	Entire cohort (*N* = 175)	Training cohort (*N* = 124)	Validation cohort (*N* = 51)
Age	< 70	66 (37.7%)	41(33.1%)	25(45.1%)
	≥ 70	109 (62.3%)	83(66.9%)	26(54.9%)
Gleason sum	7	34 (19.4%)	20 (16.1%)	14 (27.5%)
	8	65 (37.2%)	45 (36.3%)	20 (39.1%)
	9	62 (35.4%)	46 (37.1%)	16 (31.4%)
	10	14 (8.0%)	13 (10.5%)	1 (2.0%)
T-stage	T2	24 (13.7%)	16(12.9%)	8(15.7%)
	T3	66 (37.7%)	44(35.5%)	22(43.1%)
	T4	85 (48.6%)	64(51.6%)	21(41.2%)
N-stage	N0	64 (36.6%)	46(37.1%)	18(35.3%)
	N1	111 (63.4%)	78(62.9%)	33(64.7%)
Metastases	M0	26 (14.9%)	22 (17.7%)	4 (7.8%)
	Low	115 (65.7%)	82 (66.1%)	33 (64.7%)
	High	34 (19.4%)	20 (16.2%)	14 (27.5%)
t-PSA (ng/dl)	< 50	31 (17.7%)	11 (8.9%)	20 (39.2%)
	50–100	37 (21.1%)	37 (29.8%)	0 (0.0%)
	> 100	107 (61.2%)	76 (61.3%)	31 (60.8%)
CSRP1	High	107 (61.1%)	73(58.9%)	34(66.7%)
	Low	68 (38.9%)	51(41.1%)	17(33.3%)
PFS (months)	Median [min-max]	12 [3–65]	11.5 [3–65]	13 [3–38]

Abbreviations: PFS: progression free survival

For nomogram establishment and validation, 175 patients were randomly assigned to training set of 124 samples (~ 7/10), and internal validation set of 51 samples (~ 3/10). Detailed characteristics in the cohorts were summarized in Table 1. To investigate the independent risk factors for HSPC progression, we performed multivariable Cox proportional hazards regression analysis including age, PSA, Gleason sum, Clinical TNM staging information and CSRP1 expression in the training set (Fig. 7B). Specially, the ideal cutoff values for age were 70 (years) determined using X-tile software v3.6.1 (Yale University). Anymore, patients in this study with visceral and/or at least four bone metastases were classified as high-volume to distinguish them from the low-volume and non-metastases (M0). By multivariate Cox regression analysis, a nomogram integrated with the age, Gleason sum, metastases and CSRP1 expression was established (Fig. 7B, C). The concordance index (C-index) in the training and validation set were 0.74 (95% confidence interval (CI), 0.70–0.78) and 0.78 (95% CI, 0.73–0.83), respectively. The calibration plots (Fig. 7D, E) in the training and validation set both showed that the nomogram performed well in the individualized prediction of HSPC progression. Through nomogram modeling, all patients in this study were turned into a single risk score and were separated into high-risk and low-risk groups, with 48.6% (*n* = 85) in the high risk group and 51.4% (*n* = 90) in the low risk group. The K-M survival curves (Fig. 7F) revealed that the PFS of patients in low-risk group (median: 20 months) was significantly better compared with that in high-risk group (median: 7 months, *P* < 0.0001). In addition, Gleason sum and metastases volume were now clinically applied for the risk classification of metastatic HSPC. To show the predication ability of the nomogram, we constructed a clinical model based on Gleason sum and metastases in this study. The C-index of clinical model in the training and validation set were 0.66 (95% CI, 0.61–0.71) and 0.74 (95% CI, 0.67–0.81), respectively, which were all lower than those of nomogram model. The receiver operating characteristic (ROC) analysis in training and validation set showed that the area under the curve (AUC) values of nomogram for predicting the progression-free survival (PFS) were all higher than those of the clinical model (Supplementary Fig. 2A, B). The decision curves showed that the clinical effectiveness of the nomograms is better than that of clinical model within the actual threshold probability range (Supplementary Fig. 2C, D).

## Discussion

In this comprehensive study, we aimed to validate the role of CSRP1 in the progression from HSPC to CRPC. To achieve this, we employed a multiomics approach that integrated results from database analyses, in vitro experiments, and in vivo models. Our initial investigation involved analyzing proteomic data from PCa biopsy samples, as well as mining public databases. Through these efforts, we identified CSRP1 as a pivotal protein associated with the transition from HSPC to CRPC. Subsequently, we conducted a series of in vitro experiments which included assessing proliferation, apoptosis, and migration capacities of prostate cancer cells. These experiments demonstrated that lower expression levels of CSRP1 tend to enhance tumor cell proliferation, inhibit apoptosis, and promote migration. Moreover, to confirm our findings under more physiologically relevant conditions, we performed in vivo experiments using nude mouse xenograft models. The results further supported the notion that low CSRP1 expression is conducive to accelerated tumor growth. Finally, to clinically validate the association of CSRP1 with the progression of HSPC to CRPC, we carried out IHC staining on pathological sections from patients with long-term clinical follow-up data. The IHC analysis revealed a close correlation between CSRP1 expression levels and the development of CRPC, thus consolidating our hypothesis that CSRP1 plays a critical role in prostate cancer progression.

The CSRP family is a group of proteins characterized by the presence of LIM domains, which are double zinc-finger motifs that often mediate protein-protein interactions and have been implicated in various regulatory processes essential for development, cellular differentiation, gene regulation, cell growth, and somatic differentiation [16]. CSRP1, a member of this family, is a protein-coding gene with known roles in pathways such as Metal ion SLC transporters and Cellular responses to stimuli. Its function has also been linked to RNA binding based on GO annotations [17]. Notably, CSRP1 has been recognized as a tumor suppressor in several types of cancers including colorectal cancer [18, 19], and cholangiocarcinoma [20]. In our study, we present novel findings revealing that low expression levels of CSRP1 facilitate the progression of HSPC to CRPC. This discovery was substantiated through an analysis of a cohort of HSPC patients. Furthermore, integrating KEGG and GO analyses, we found that CSRP1 is part of a module closely associated with cell adhesion molecules. Given that the progression of HSPC has been previously tied to invasion-related genes like intercellular cell adhesion molecule-1, it is plausible that CSRP1 may modulate the invasive capacity of prostate cancer cells, thereby influencing the transition from HSPC to CRPC [21].

While numerous studies have focused on identifying biomarkers for HSPC, there is a significant gap in validating these findings in real-world populations [22, 23]. Our study not only reaffirmed the functional role of CSRP1 through routine in vivo and in vitro experiments but also provided clinical validation via IHC staining of pathological sections from HSPC patients with long-term follow-up data. Recent research has shown that the time to progression from HSPC to CRPC varies widely among patients receiving standard ADT [24]. Multiple phase-III trials underscore the importance of early identification of HSPC patients at high risk of rapid disease progression to enable prompt initiation of targeted therapies, thus improving patient outcomes [25]. Two pivotal studies have attempted to classify metastatic HSPC patients based on their risk profiles. The CHAARTED trial categorized patients with visceral metastases or four or more bone metastases as ‘high-volume’ versus ‘low-volume’ patients who did not meet these criteria. The study demonstrated that high-volume patients benefit significantly from combined ADT and docetaxel treatment, while low-volume patients showed adequate response to ADT alone. On the other hand, the LATITUDE trial identified patients with two or more high-risk features (three or more bone metastases, visceral metastases, or ISUP grade 4) as high-risk, revealing that these patients had improved survival rates when treated with abiraterone acetate plus prednisone. However, despite these classifications, many patients labeled as low-risk or low-volume according to the CHAARTED and LATITUDE criteria experienced rapid progression to CRPC. In our cohort, most patients would have been classified as such, yet they showed a notably swift median time to progression of nine months. This highlights the inadequacy of relying solely on clinical imaging or M stage for patient stratification.

Indeed, the results of our study point to CSRP1 IHC as a promising candidate for inclusion in risk assessment and individualized treatment planning for patients with HSPC. The expression levels of CSRP1 could provide valuable information that goes beyond conventional staging systems, helping clinicians to identify those patients who are at higher risk of rapid progression to CRPC. Incorporating CSRP1 IHC into routine clinical practice may enable more accurate predictions about patient prognosis and guide tailored interventions. For instance, patients with low CSRP1 expression might benefit from early initiation or intensification of therapies known to slow disease progression or improve survival outcomes. This targeted approach would align with the growing trend in precision medicine, where treatment decisions are based on the molecular profile of the tumor. Future studies should further validate these findings across larger patient cohorts, assess the predictive power of CSRP1 expression alongside other established biomarkers, and explore its potential role in guiding therapeutic choices in HSPC management. Ultimately, this could lead to improved patient care and better overall outcomes by ensuring that high-risk patients receive the most effective treatments as early as possible.

Our study’s findings are indeed significant, but it is important to acknowledge its limitations. The primary constraint was the small sample size derived from a single medical center, which may affect the generalizability of our results to a broader patient population. Furthermore, all participants in our research were treated with standard ADT combined with bicalutamide or flutamide. In contemporary clinical practice, however, treatment regimens for advanced prostate cancer are evolving rapidly. ADT in combination with these antiandrogens is increasingly being supplanted by more aggressive approaches such as chemotherapy coupled with second-generation hormone therapies like abiraterone acetate or enzalutamide. Therefore, to substantiate our observations and explore the potential application of CSRP1 expression in guiding personalized therapeutic strategies, there is an urgent need for larger, multicenter studies that encompass diverse patient populations and reflect current treatment standards. These future studies should include comparisons across different treatment modalities to discern how varying therapeutic interventions interact with CSRP1 expression levels and influence disease progression and response rates. This will enable a clearer understanding of the benefits associated with various treatment strategies when tailored to specific patient subgroups based on molecular biomarkers like CSRP1.

## Electronic supplementary material

Below is the link to the electronic supplementary material.

Supplementary Material 1

Supplementary Material 2

Supplementary Material 3

## Data Availability

The mass spectrometry proteomics data have been deposited to the iProX with the dataset identifier IPX0007011000 (the data will be publicly released upon publication).
