# Supplementary material for: Identification of CSRP1 as novel biomarker for hormone-sensitive prostate cancer by the combination of clinical and functional research
Source: Cancer Cell Int. 2025 Feb 24;25:65. doi: 10.1186/s12935-025-03708-y (PMC11849366; doi:10.1186/s12935-025-03708-y)
Supplement: Supplementary file 1 — Supplementary Material 1 [file 12935_2025_3708_MOESM1_ESM.docx]

**Supplementary Figure 1.** (A) Differential expression gene of GSE35988. Blue dots indicate down-regulated and red dots indicate up-regulated. (B) Analysis of the scale-free fit index for various soft-thresholding powers. (C) Analysis of the mean connectivity for various soft-thresholding powers. (D) Stable CSRP1 overexpression was confirmed by qRT-PCR in LNCaP cells;

**Supplementary Figure 2.** **Validation of predictive capacity of the nomogram in the training and testing set.** (A) Comparing ROC curves of the nomogram and clinical model for 0.5-year, 1-year, 1.5-year and 2-year PFS. (B) Comparing the time-dependent decision curve analysis for the clinical benefit of the nomogram and clinical model.

**Supplementary Table 1.** **Clinical characteristics of three CRPC patients.**

| Patient ID | CR1 | CR2 | CR3 |
| --- | --- | --- | --- |
| Age at PCa diagnosis, years | 83 | 65 | 81 |
| PSA at diagnosis, ng/mL | 86.7 | 4595.0 | 715.4 |
| Clinical TNM stage | T2N0M1c | T3N1M1c | T4N1M1b |
| Biopsy Gleason score | 5+4 | 5+4 | 4+4 |
| Time to CRPC, months | 25 | 20 | 7 |

Note. PCa: prostate cancer; CRPC: castration‐resistant prostate cancer; PSA: prostate‐specific antigen;
