# Supplementary figures and images for: Identification of CSRP1 as novel biomarker for hormone-sensitive prostate cancer by the combination of clinical and functional research
Source: Cancer Cell Int. 2025 Feb 24;25:65. doi: 10.1186/s12935-025-03708-y (PMC11849366; doi:10.1186/s12935-025-03708-y)

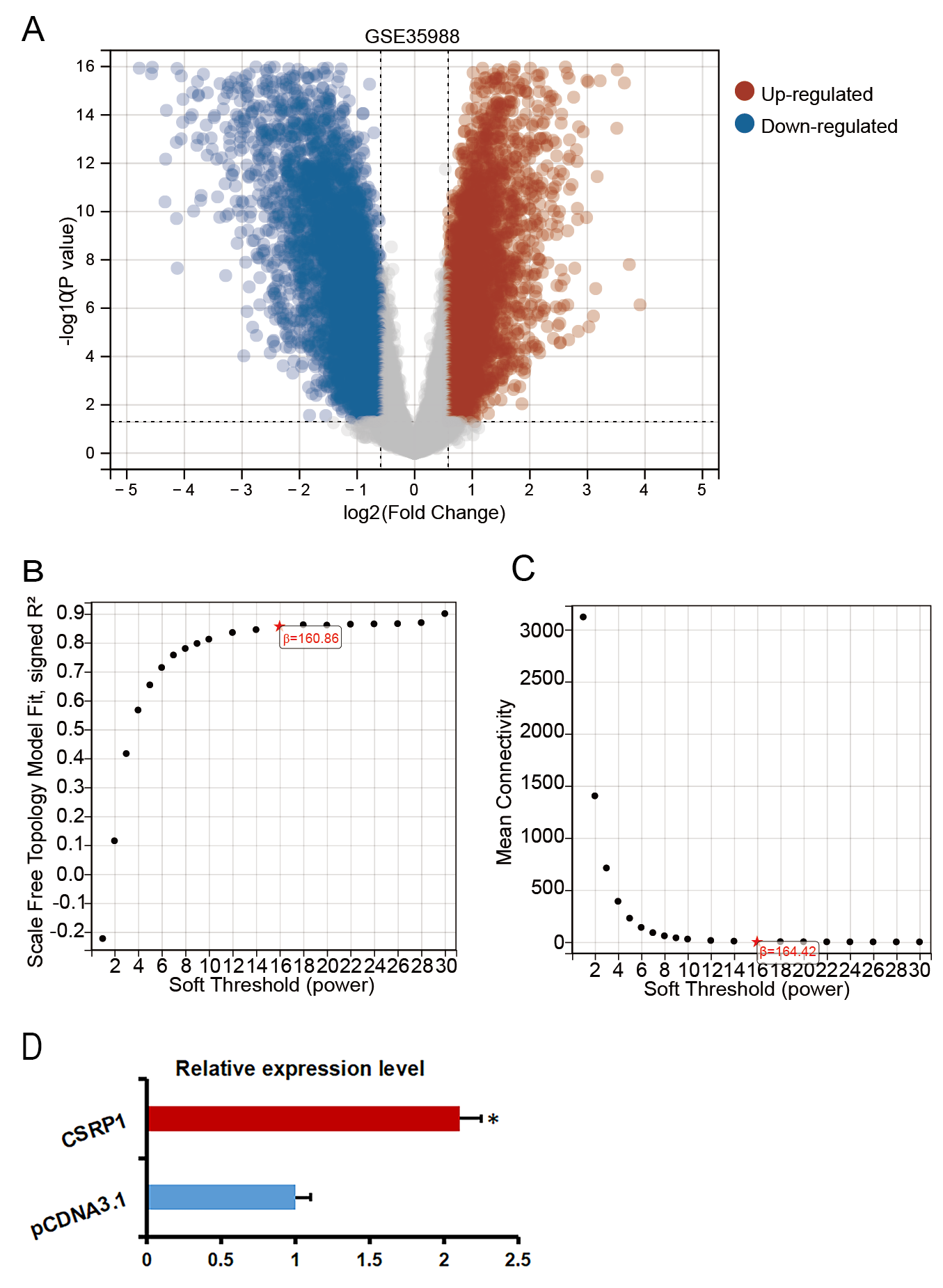

Supplement: Supplementary file 2 — Supplementary Material 2 [file 12935_2025_3708_MOESM2_ESM.tif]

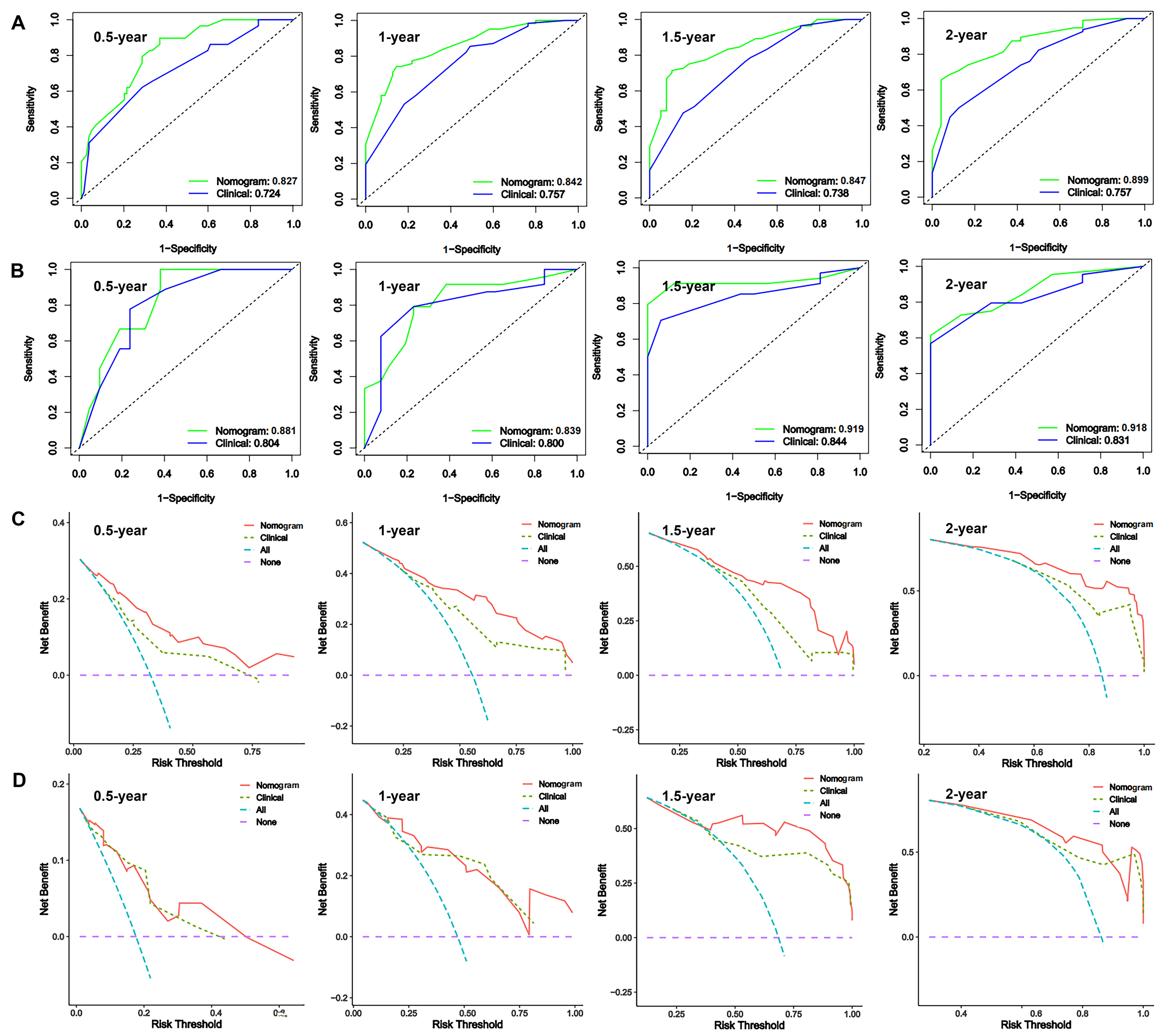

Supplement: Supplementary file 3 — Supplementary Material 3 [file 12935_2025_3708_MOESM3_ESM.tif]
